# Pathology of Asphyxia

**Published:** 1853-07

**Authors:** Thomas Johnson

**Affiliations:** Late Professor of Anatomy in the University of Virginia


*On the Pathology of Asphyxia.* By Thomas Johnson, M. D., late Professor of Anatomy in the University of Virginia.

There is probably no term whose literal meaning conveys so little idea of the pathological conditions classed under it, as asphyxia. Syncope is accompanied by a cessation of pulse; yet there is a marked difference between the two affections, which is obvious to the most superficial observer.

Physiologists are agreed, that of all the injurious accumulations in the blood, that which takes place with the greatest rapidity, is carbon, or carbonic acid. To separate this noxious ingredient from the circulating mass, and to supply it with the vivifying principles of the atmosphere, is a work which must go on unceasingly. The largest gland in the body of vertebrata, though subservient to other important functions, is also actively separating carbon from the blood, by throwing it into innocuous combinations, thereby assisting the pulmonary organs in no inconsiderable degree. But it is evident, any circumstance which interrupts the atmospheric air into the lungs, increases the carbon in the blood; at the same time, this vital fluid, by the exclusion of oxygen, is deprived of a stimulus essential to the vital actions of the general tissues. If there is not a due balance kept up by a normal respiration, between that act and circulation, animal heat, nutrition, secretion, absorption, innervation, in a word, every vital action, feels the influence.

There are two general causes of the asphyxia; the first, mechanical, comprehending such agents as prevent the æriform medium in which we live from entering the air-cells of the lungs; and the second, chemical, including those gases which are destructive of the respiratory act, whether from the deficiency of oxygen in their composition, or from their positively deleterious properties.

It is a well established fact, that reptiles and the invertebrata, as well as hybernating mammals, require less oxygen to support life than the birds and the mammalia in general. The cetaceæ, though mammals, are enabled to remain under water an hour or more, because their arteries and veins lie in contact with large diverticula, filled with red blood, which is absorbed and taken into the circulation from these reservoirs during submersion. The respiratory function in man ceases in less than five minutes after the complete exclusion of atmospheric air, and the circulation in less than ten minutes; but the habit may prolong this period, as is incontestibly proven by Ceylon pearl divers. Reanimation of still-born infants has been known to take place half an hour after the resuscitating means were resorted to.*

Too frequent respiration of the same air as surely produces asphyxia as the breathing of carbonic acid, though not so quickly.—The horrors of the Black Hole of Calcutta, in 1756, must be familiar to every one, where 146 persons were confined for a night, in a badly ventilated room, only 18 feet square. The next morning, only 23 of the prisoners were alive, and it is said, (mark the observation.) most of these subsequently perished of "putrid fever."

Whether what are termed *Zymotic* diseases depend in reality upon a fermentable material or not, may be disputed; but certain it is, that an inferior degree of pulmonary and cutaneous exhalation is a most potent predisposing cause of choleriform and typhoid affection. When air is confined and frequently respired, it becomes loaded with carbonic acid and putrescent exhalations from the body, and is finally offensive to the smell. How can we doubt that such

* Twelve years ago, I attended a negro woman, who was delivered of twins. The last child was still-born, and I tried fifteen or twenty minutes to resusciate him, unsuccessfully. An old negress then applied her mouth to the child's, I holding his nose, and at regular intervals pressing the abdomen and thorax, so as to imitate the respiratory action of these parts. In five minutes the child cried lustily.

For interesting cases of this kind, and those from drowning, see Mare, *Manuel d'Autopsie Cadaverique Medico-Legale*, p 152, et seq.

an atmosphere is a cause of zymotic diseases? Whilst complete oxidation of effete matters eliminates them in the form of carbonic acid and water, leaving urea and other highly oxidized products to pass off by the kidneys, an imperfect oxidation will convert them into peculiarly offensive products, such as those which characterize fœcal excretions and imperfectly formed bile.*

The reports of boards of health uniformly attribute the spread of zymotic diseases to overcrowding and insufficient ventilation. We observe the prevalence of these affections in prisons, in ships, in camps, poor-houses, factories, &c. It is in such situations that cholera, asphyxia, and typhoid fever rage with the most terrible malignancy. The same effect is observed when the atmosphere is contaminated by the evolution of carburetted hydrogen from marshy soils. The history of the cholera at Kurrachee in Scinde, where an army of 6380 men was decimated in an incredible short space of time, strongly confirms this view.† Watchfulness, the influence of warm climates, and prolonged muscular exertions, by well known physiological laws, cause not only imperfect aeration of the blood, but the most noxious cutaneous exhalations.

From these considerations, it will appear that the subject of deficiency of proper respiratory material is of the deepest hygienic interest to the public generally, as well as to the pathologist.

Asiatic cholera has been most appropriately termed cholera asphyxia, for partial asphyxia is a most prominent and invariable symptom of the disease. The blood drawn from the vein of a cholera patient is dark, thick, and grumous; and the arterial blood is as black as ordinary venous blood. This is precisely the condition of the blood in asphyxia from submersion or strangulation. It is to be remembered that the functions of the liver, a powerful decarbonizing organ, are immediately suspended in severe cases of cholera.

The phenomena of asphyxia can be best observed where they gradually occur, as in the partial obstruction of the air-passages, produced by pneumonia and similar affections; for, where they succeed each other rapidly, as in strangulation, they do not admit of accurate observation. Dr. Carpenter very properly divides them into three stages or degrees. The first commences with an increase of the *besoin de respirer*, which may increase to a most distressiug extent. Involuntary and most powerful efforts of the respiratory muscles respond to the wants of the system, and if aeration does not occur, the agony becomes intense, succeded by vertigo and insensibility.

When this insensibility is complete, the respiratory movements become irregular and convulsive, the contenance is livid, the nos-

---

* Carpenter. *Principles of Physiology, p.* 539.

† See *British and Foreign Medico-chirurgical Review.*

trils dilate, the lips are of a deep purple, the veins turgid, and the eyes seem starting from their sockets. This is the second stage. The pulse at the wrist is now almost extinct, and the heart pulsates languidly. Soon the animal functions are suspended; no muscular movements are observed, and the sphincters begin to relax.

In the third stage the irritability of the voluntary muscles is gone, the individual falls powerless to the ground, but still the heart contracts feebly, though unable to force the blood into the systemic arteries. The general surface now becomes livid; a characteristic violet hue of the hands and feet is observed; purple spots appear upon other parts of the surface; cadaveric rigidity takes place tardily after the organic functions have been entirely suspended.

It is an indisputable fact, that the change from venous to arterial blood, is effected in the pulmonary capillaries, and there the sagacity of Haller fixed the seat of asphyxiation, though he was probably wrong in supposing the stagnation to depend merely upon a mechanical impediment to the motion of the lungs. If an animal is asphyxiated by breathing the irrespirable gases, the air-cells remain open, and there is no direct obstruction to the free flow of blood through the pulmonary structure; yet, here we observe nearly the same phenomena as if it had been submerged or strangulated. Nor does death, under the circumstances of asphyxia, proceed from the want of the arterial stimulus in the left side of the heart; for Bichat has experimentally proven that the presence of venous blood, is sufficient to keep up the action of both sides of the heart: besides, the facts connected with hybernating animals, whose blood is almost purely venous, during hybernation, is opposed to such a theory. Bichat ascribed the cessation of the circulation to diminished irritability of the heart, resulting from the deleterious effect of venous blood upon its vital properties, an effect which he proved this fluid to exert on the excitability of the nervous system.* That the circulation of venous blood upon the nervous centres, acts powerfully in arresting the circulation, I cannot doubt, notwithstanding the experiments of Dr. Kay prove that the circulation of venous blood through the muscles is not destructive of *their* irritability.† But it must be borne in mind, that an interruption of the chemical changes, or aeration in the pulmonary capillaries, is the primary cause which leads to the stagnation or engorgement in these capillaries; hence, if these chemical agents be renewed before organic life becomes extinct,

* See Carpenter, Legallios and Bichat.

† See *Recherches sur le retablissement de l'irritabilite musculare*, by Dr. Brown-Sequard. It is there proven, from experiments on criminals, that muscles affected with cadaveric rigidity, regain their irritability upon the injection of venous blood into their vessels.

the vital actions may be renewed in the organism. The right side of the heart, according to all observation, in cases of asphyxia, will soon cease to act from over-distension of venous blood which accumulates in it; whereas, the left side speedily ceases to act from a deficiency of blood, the pulmonary veins having ceased to afford a supply to it. These opposite conditions of the heart, are always found to exist where death has resulted from slow asphyxia.

All injuries and diseases which terminate in coma, occasion death by asphyxia, a principle which leads to important practical results; for, if there be no organic lesion of the nervous centres, and the stupor is temporary, life may be preserved by a long-continued artificial respiration. In cases of narcotic poisons, and convulsions in children, this practice has been followed by successful results, and as Sir Benjamin Brodie remarks, is now one of the established facts of our profession. Observation of the progress of the symptoms of typhoid fevers, induces me to believe, that the coma and organic lesions of the brain, which are developed in their course, is the result of partial asphyxiation; for, where post-mortem examinations show lesion of the brain, or its meninges, dyspnœa has been a prominent symptom of the case, and has uniformly preceded cerebral disturbance.

Strangulation, as every one knows, is a violent compression of the neck by a ligature, or other means, and whether it be by suspension, the garotte, or otherwise, the impediment to respiration may be partial or complete. If the ligature is only applied to the trachea, death occurs from asphyxia; but if the jugular veins be compressed, cerebral congestion or apoplexy ensues. Any one who will reflect upon the functions of the parts involved, and the nature of the mechanical application, must be convinced that in strangulation, both of these conditions usually occur together. Casper observed, that in 106 cases of strangulation, apoplexy took place in nine; asphyxia alone in fourteen; both conditions, in sixty-two; while the remaining sixteen cases were not examined.

A vulgar prejudice exists, that hanging by the neck often produces dislocation or fracture of the cervical vertibræ. It is remarkable that neither Casper, Remer,* or Fleishmann, mention any such occurrence. When we consider the anatomical structure of the neck, and reflect upon the number and strength of the muscles and ligaments which firmly bind the cervical vertebræ together, we would naturally infer dislocation or fracture to be a very rare accident in death by suspension. Although I have had some opportunities for examination of cases of death from suspension, I have never seen either of the conditions just referred to.†

* *Annales of Hygiene.*

† Persons who have been recovered from attempts at strangulation, suppose it to be a very easy mode of death. The symptoms which precede insensibility are, according to such testimony, rather pleasurable (!) than painful. A lady, who

In death by submersion, water is often found in the air-passages of the lungs, acting as a mechanical obstruction, and affording a greater or less impediment to the entrance of air to the cellular tissue of these organs; but this cannot properly be considered a proximate cause of death, for, frequently, autopsies show this condition to be absent, and it would require a considerable quantity of fluid to entirely impede the pulmonary circulation, in the manner described by Dr. Southwood Smith. Nor does the partial collapse of the lungs, so much insisted upon by some authorities, even when it exists, account for the rapid death which supervenes upon complete immersion.

One of the consequences of asphyxia, (though as we have already shown by means of a primary one,) is congestion of the cerebral veins. This occurrence is pretty uniform in death by drowning, but it cannot be regarded the proximate cause of death, inasmuch as it is only one of the consequences of that stagnation of blood in the lungs, which affects so injuriously all the vital offices of the system. However, that death is sometimes produced exclusively by congestion, in cases of submersion, I am not disposed to deny.

The principal cause of death in the greater number of cases of submersion, is from asphyxia, induced by the supply of atmospheric air being cut off from the lungs, thus preventing the æration of the blood in the air-cells of those organs. In the act of drowning, most of the atmospheric air is expelled from the lungs before final sinking under the water, especially if that medium be cold, but still some air remains in the air-cells and bronchi. This soon becomes contaminated and is rendered unfit for respiration by the evolution of the deleterious carbonic acid gas.

In simple asphyxia, rapidly induced, the general lividity of the body is quite characteristic; though it may be absent in cases of drowning. When the patient has been strangled, deep livid spots, resembling ecchymosis, are usually observed upon the face, neck and upper part of the chest. The face has a distressed expression, the eyes are prominent and the pupils dilated. The right side of the heart and the great veins are congested with a semi-fluid black blood, whilst the left side is comparatively empty. The large organs, as the spleen and liver, are engorged with venous blood, and so are the mucous membranes, which sometimes exhibit ecchymoses. This pseudo-morbid appearance on the mucous surface of the stomach of a subject recently hanged, was mistaken by Yellowby for the characters of inflammation, and this case has been, tried the trick of suspending herself by the neck from a bed-tester, says, the first sensations are very much like those produced by taking chloroform. In her case, she experienced a brief vertigo, some pain on the top of the head, pain of the epigastrium, tinnitus aurium, and flashes of light before the eyes; all of which were soon relieved by complete insensibility.

singularly enough, appealed to, by the opponents of Broussais, as a refutation of his doctrines of gastritis. To the practised eye, ecchymosis no more resembles inflammation than it does mortification.

The brain, the root of the tongue, the mucous membrane of the bronchi, and the lungs, are discoloured by venous turgescence. The lungs themselves, says Carpenter, in his admirable Essay on Asphyxia, if not previously diseased, are generally distended, and expand so as to meet over the pericardium. When exposed to view, they present a dark brown, sometimes almost blackish hue externally; but their parenchyma exhibits a redder tint when cut into. The engorgement is here in the pulmonic arterial system; but it is occasioned by the accumulation of venous or black blood, of which, large, dark, thick drops flow out when incisions are made in the substance, and slight pressure employed.

The anatomical characters of strangulation by hanging have been well summed up by Taylor, (*Medical Jurisprudence*, vol. 1, p. 165.) These are lividity and swelling of the face, especially of the lips, which appear distorted. The eyelids are swollen and of a bluish color; the eyes red, projecting forwards, and sometimes forced out of the orbital cavities; the tongue enlarged, livid, and compressed between the teeth, and frequently protruded. A sanguineous froth about the lips and nostrils. A deep and ecchymosed impression around the neck, indicating the course of the cord, the skin sometimes being excoriated; laceration of the muscles and ligaments of the hyoideal region; laceration or contusion of the larynx, or upper part of the trachea. There are, commonly, circumscribed ecchymosed patches, varying in extent, about the upper part of the trunk, with deep livid discoloration of the hands and feet, and a violet tint of the nails. The fingers are generally much contracted, or firmly clenched, The urine, the fæces, and seminal fluid are sometimes involuntarily expelled at the moment of death. The body is, *cœteris paribus*, a much longer time than usual in parting with its heat. Most of these signs are observed on the bodies of persons who have been hanged, but it is remarkable how many of them may be absent.* The countenance of suicides frequently presents the traits of the most undisturbed placidity, whilst the countenance of criminals, who have been executed, or of persons who have been murdered, exhibits great distress or even distortion.

"But, see, his face is black, and full of blood,
His eyeballs farther out than when he lived,
Staring full ghastly like a strangled man:
His hair uprear'd, his nostrils stretched out with struggling;
His hands abroad displayed, as one who gasp'd
And tugg'd for life, and was by strength subdued."
(*Henry VI., Pt. II., Act 3, Scene 2.*)

* See a remarkable case of suicide related by Esquirol, *Maladies Mentales*, Tome II, p. 844.—[Ed.

In cases of submersion, where simple asphyxia has been the result, the face is either pale, or a slight livid tint is observed; but the hands and feet are usually discolored. As Carpenter remarks, "the appearance of the surface will greatly depend upon the duration of the immersion, and upon the length of time during which the body has been subsequently exposed to the air." The pupils are dilated, and the eyelids half open; a mucous froth about the mouth and nostrils; the tongue pushed forward against the teeth. The bronchial mucous surface is generally injected, but the air-passages do not usually contain more than a spoonful of water. The lungs are gorged with black fluid blood. If the body has been long immersed, the stomach becomes filled with water.

When syncope has been the cause of death, in drowning, the surface is pale and almost in a natural condition. The trachea sometimes contains a little water, but no froth; the lungs are sometimes collapsed, but never preternaturally distended. The brain is seldom apoplectic, though there may be some cerebral engorgement.

It is scarcely necessary to observe, that the first thing to be done in the treatment of asphyxia, is to remove the cause, since, if it continues to exist, all efforts at relief must be unavailing. When the respiratory function has not entirely ceased, the patient, usually, is easily restored by free exposure to the air, and keeping the surface of the body moderately warm; but where these have been suspended, more energetic measures must be resorted to. As artificial respiration is the most powerful means for the restoration of suspended animation, it must be employed without delay. The chest and abdomen should be compressed so as to diminish the cavity of the thorax, thereby expelling as much as possible the contents of the air passages, and then the thorax is allowed to regain its natural size by its own elasticity. This should be repeated 15 or 20 times a minute. Whilst this process is in operation, the lungs should be inflated to ærate the blood which has become stagnant in the pulmonary capillaries.

In addition to artificial respiration, stimulating agents are to be employed. Artificial heat, not exceeding 98°, should be applied to the surface of the body. The warm bath and warm applications to the epigastrium are of great importance, especially after submersion, in restoring the natural temperature of the body. Frictions with warm flannel and rubefacients will be found useful, after the warm bath. Moderately stimulating fluids may be injected into the stomach or rectum with advantage. Electric shocks through the region of the heart has been known to excite it to action, even after the case has been abandoned as hopeless. (See Dr. Ure's interesting cases, in the Glasgow Med. Jour.)

Where the jugular veins are much distended, they should be emptied by an incision with a lancet, thereby relieving the brain and heart of an oppressive engorgement. However this remedy should be applied with great caution, for reasons which must be obvious to every practitioner.

The consideration of asphyxia in relation to hygiene and medico-legal questions is of great importance, but as these are extensive subjects, I purpose recurring to them at a future period.

